# Surgical Management of Recurrent Brain Metastasis: A Systematic Review of Laser Interstitial Thermal Therapy

**DOI:** 10.3390/cancers14184367

**Published:** 2022-09-08

**Authors:** Mark A. Damante, Joshua L. Wang, J. Bradley Elder

**Affiliations:** The Ohio State University Wexner Medical Center, Department of Neurosurgery, Columbus, OH 43210, USA

**Keywords:** brain metastasis, laser interstitial thermal therapy, LITT, recurrent brain metastasis, intracranial progression, craniotomy

## Abstract

**Simple Summary:**

Advances in surgical techniques, radiation therapy and systemic therapies have greatly improved survival among patients with brain metastases. Prolonged survival inevitably leads to an increased incidence of local disease recurrence. The management of recurrent brain metastases is not uniform. Treatment modalities include re-resection, irradiation, alternate systemic therapy regimens and, more recently, laser interstitial thermal therapy. Laser interstitial thermal therapy, or LITT, may offer an effective surgical alternative to traditional craniotomy for resection, particularly in the setting of a patient that is unable to tolerate open surgery or with a deep-seated brain metastasis that is not amenable to surgical resection. This review aims to evaluate the available literature regarding the use of LITT specifically for recurrent intracranial disease.

**Abstract:**

The incidence of recurrent metastatic brain tumors is increasing due to advances in local therapy, including surgical and radiosurgical management, as well as improved systemic disease control. The management of recurrent brain metastases was previously limited to open resection and/or irradiation. In recent years, laser interstitial thermal therapy (LITT) has become a promising treatment modality. As systemic and intracranial disease burden increases in a patient, patients may no longer be candidates for surgical resection. LITT offers a relatively minimally invasive option for patients that cannot tolerate or do not want open surgery, as well as an option for accessing deep-seated tumors that may be difficult to access via craniotomy. This manuscript aims to critically review the available data regarding the use of LITT for recurrent intracranial brain metastasis. Ten of seventy-two studies met the criteria for review. Generally, the available literature suggests that LITT is a safe and feasible option for the treatment of recurrent brain metastases involving supratentorial and cortical brain, as well as posterior fossa and deep-seated locations. Among all studies, only one directly compared craniotomy to LITT in the setting of recurrent brain metastasis. Prospective studies are needed to better elucidate the role of LITT in the management of recurrent brain metastases.

## 1. Introduction

### 1.1. Rationale

Improved access to advanced radiographic technology has led to an increase in the detection of brain metastases [1]. Further, the improvement of surgical techniques, radiation strategies and systemic therapies to treat brain metastases has led to significantly longer survival and subsequently increased need to treat local central-nervous-system disease recurrence [2]. The surgical resection of brain metastases has become an important component of the treatment of solitary tumors. In the 1990s, Patchell et al. and Vecht et al. suggested that resection with adjuvant radiation provided superior overall and progression-free survival and functional independence for patients compared with radiation alone [3,4]. Despite successful surgical resection, brain metastasis recurs in 20–40% of patients treated with surgery alone [3,5,6,7]. Intracranial recurrence is subdivided into local recurrence, defined as regrowth at the site of a previously resected or irradiated lesion, and distant recurrence, defined as new sites of disease in the brain that develop separately from a treated site. The risk of local recurrence is associated with the size of the lesion, subtotal resection or residual microscopic disease [8]. The best practice for the management of recurrence is poorly understood and ultimately depends on the decision of a multidisciplinary discussion among the neurosurgeon, radiation oncologist and medical oncologist. The management of recurrence can be categorized into surgical and non-surgical options. Surgical intervention includes both open resection via craniotomy and laser interstitial thermal therapy (LITT), whereas non-surgical options include radiotherapy or adjustments in the systemic therapy regimen.

### 1.2. Objectives

The following manuscript aims to evaluate the surgical options, including LITT, for recurrent brain metastases, as well as to provide a systematic review of the use of laser interstitial thermal therapy for recurrent brain metastases.

## 2. Materials and Methods

**Figure 1:**
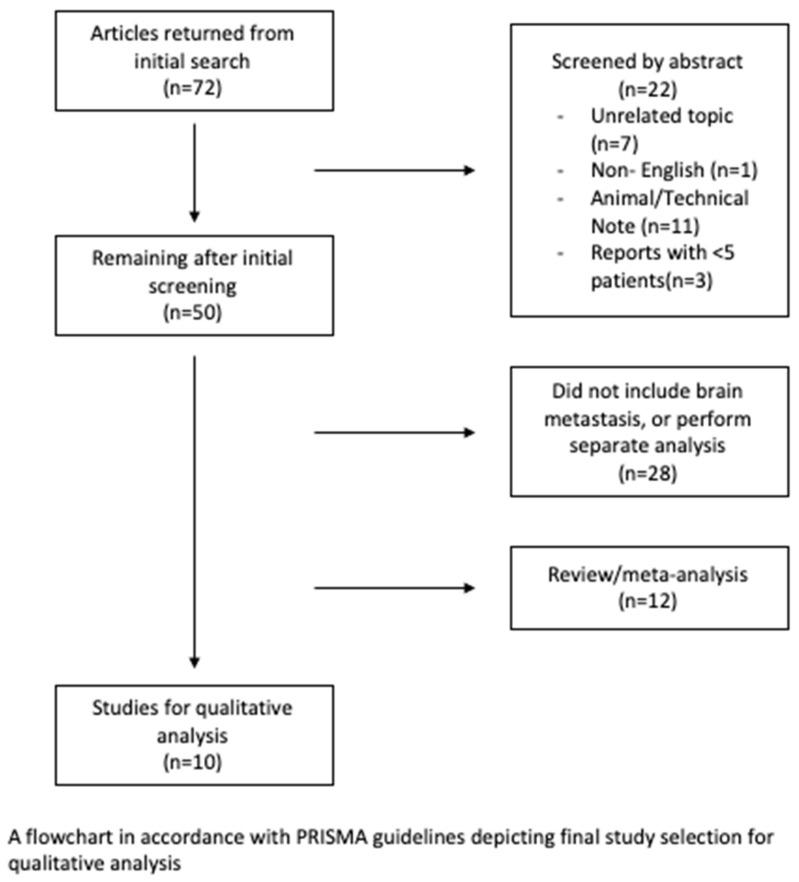
PRISMA guideline flowchart.

A systematic review of the current literature regarding the management of recurrent brain metastasis with laser interstitial thermal therapy was conducted according to PRISMA guidelines (Figure 1). Articles were selected from the MEDLINE (PubMed) database. The following search string was used: (“laser interstitial thermal therapy” OR “LITT” OR “laser ablation”) AND ((“recurrent” OR “progressive” OR “progression”) AND (“brain” OR “cerebral” OR “intracranial”) AND (“metastases” OR “tumor”)). The search returned 72 articles. After the evaluation of the search results against the inclusion criteria, 10 articles remained for the qualitative analysis. Several studies were multicenter analyses; therefore, they may have had unavoidable overlap in patients, although likely minimal and infrequent.

### Eligibility Criteria and Study Selection

Peer-reviewed articles evaluating LITT in the management of recurrent metastatic brain lesions published between 1 January 2002 and 1 May 2022 were reviewed. Search criteria were limited to human studies written in English (*n* = 11 animal studies/technical notes, *n* = 1 non-English). Review articles without the inclusion of the authorships’ institutional experience were not included (*n* = 12). Case reports were included only if ≥5 patients were included (*n* = 3 reporting <5 patients). Many articles reviewed included a heterogenous cohort of radiation necrosis, recurrent brain metastasis and primary brain tumors or did not include recurrent brain metastases all together. Such articles were not included unless the report interpreted metastatic disease results independently from non-metastatic disease (*n* = 28).

## 3. Results

Ten studies met the inclusion criteria for this review (Table 1). These studies included 412 total lesions, of which 303 (73.5%) were identified as recurrent brain metastases. The patients were followed for an average of 6.47 months. Lesion locations (if specified) included 112 supratentorial, 62 infratentorial and 14 deep-seated lesions. When progression-free survival (PFS) and overall survival (OS) were reported, the average PFS after LITT was 7.5 months, and the average OS after LITT was 21.5 months.

## 4. Discussion

### 4.1. Re-Resection for Brain Metastasis

In the initial study by Patchell et al., 12-month local control after surgery alone was 54%. In a more recent prospective trial by Mahajan et al. evaluating SRS for local failure after craniotomy, there was a 45% 12-month local failure rate [9]. It is important to note that despite advances in neurosurgical techniques and neurosurgical adjuncts to maximize the chances of the successful surgical resection of brain metastases, the rate of local failure remains unchanged, thus necessitating continued improvement in the management of recurrent disease. The decision to resect recurrent disease is dependent on the same clinical factors that are considered for the initial operation: clinical presentation, patient performance status, anatomic location and tumor histology. Sundaresan et al. was among the first to prospectively analyze a small cohort of 21 patients with recurrent symptomatic brain metastases in the setting of clinical stable extracranial disease. Re-operation made possible the recovery of the neurologic baseline, improved performance status in approximately two-thirds of the cohort and led to a median overall survival from time of re-resection of 9 months [7]. This study was one of the first to suggest that the appropriately selected patient with recurrent intracranial disease may benefit from repeat resection both from an oncologic and a functional perspective. Bindal et al. retrospectively reviewed a group of 48 patients with previously resected, recurrent brain metastases. Reoperation conferred a median overall survival of 11.5 months, which at the time of publication was comparable to the survival benefit historically afforded via initial resection [4,5]. Interestingly, 26 patients from this cohort went on to recur a second time, of which 17 underwent a third resection. In a small comparison of these 26 patients, a third resection significantly improved OS from the time of recurrence (8.6 months vs. 2.8 months; *p* < 0.0001) [5]. While these data support an aggressive surgical management of recurrence, treatment selection bias cannot be overlooked. These cohorts comprised patients with good performance status and tumors in a readily accessible anatomic location. In contrast with the open resection of recurrent brain metastases, laser interstitial thermal therapy has emerged as a surgical alternative to craniotomy, particularly among patients who may not tolerate open surgery or who have deep-seated lesions, in which an open approach to such a lesion would traverse a significant volume of healthy brain or involve dissection into deep nuclei.

### 4.2. Laser Interstitial Thermal Therapy for Recurrent Disease

Open resection remains an effective approach for the treatment of recurrent intracranial disease; however, not all recurrence is amenable to open surgery. Previously, recurrent intracranial disease in poor surgical candidates, deep-seated or eloquent areas, or diffuse metastases were limited to management by re-irradiation. In the past twenty years, laser interstitial thermal therapy (LITT) has emerged as a viable option for the treatment of recurrent disease, with similar indications and advantages provided by radiotherapy.

LITT is a minimally invasive approach to the treatment of both intracranial recurrent disease and radiation necrosis. Compared with traditional craniotomy for open resection, LITT most often involves a small incision for the placement of a single burr hole. A fiber optic probe is placed within the target lesion, and intraoperative MRI is used to both confirm the accuracy of probe placement within the target lesion and monitor the ablation. Real-time MRI-guided thermometry is used to monitor tissue thermodynamics, which ensures effective and safe thermal ablation. Standard thermal dose thresholds (TDTs) are identified intraoperatively by varying the colors associated with the temperature the encompassed tissue receives for a projected period of exposure time [10]. The NeuroBlate system (Monteris) and the Visualase (Medtronic) system use a formula to calculate thermal energy delivery. For example, the NeuroBlate system states that the outer yellow TDT signifies the thermal dose equivalent of 43 degrees Celsius for two minutes; the middle blue line indicates a thermal dose equivalent of 43 degrees Celsius for ten minutes; lastly, the inner white line indicates a thermal dose equivalent of 43 degrees Celsius for a 60 min duration. Target temperatures between 46 degrees Celsius and 60 degrees Celsius cause irreversible damage leading to cell death [11,12]. It is suggested that pathologic tissue has a higher absorption coefficient, which results in faster ablation than healthy brain parenchyma, thus favorably treating the lesion with a lower risk of damage to surrounding tissue [13]. Visualase (Medtronic) offers a similar procedure with a shorter wavelength laser, thus providing a more efficient heating of the target tissue while slightly limiting tissue penetration and ablation volumes in areas with higher cerebral perfusion. The Visualase system also employs the smallest voxel size, allowing a more precise delivery of thermal damage to be performed. Comparatively, the NeuroBlate system employs a side-firing tip, which makes possible the directionality of thermal damage, and may be observed during intraoperative MRI guidance. Neither system has been compared head-to-head and the included studies report the use of either system based on the publishing institution.

Data comparing craniotomy to LITT for the management of recurrence are lacking. Most publications are small, single-institutional experiences that aim to define the feasibility of LITT in the management of recurrence and/or radiation necrosis alone, often with an emphasis on tumors deemed inoperable due to deep or eloquent location.

Salehi et al. retrospectively reviewed 25 patients with recurrent brain metastases, of various histologies, treated with LITT [10]. Patients were followed for a median for 16 months and achieved median OS of 13.27 and median PFS of 6.3. A subset analysis divided these 25 patients in several ways, which further enhanced the understanding of the implementation of LITT. First, the authors demonstrated that a pre-procedural tumor volume greater than 5.62 cm^3^ (*n* = 11) resulted in significantly decreased PFS (*p* = 0.024, HR 2.89 (1.12–7.49)) compared to tumor volumes smaller than 5.62 cm^3^ (*n* = 12) [10]. Secondly, their group determined that the treatment area covered by the blue thermal dose threshold (TDT) line, or 43-degrees Celsius for 10 min, led to the improvement of both PFS and OS [10]. A total of 23 out of 25 patients (two lacked necessary data) were dichotomized into a > 97% coverage (*n* = 10) and a ≤ 97% coverage (*n* = 13) by the blue TDT line. When >97% coverage was achieved, a significant increase in median PFS was noted (*p* = 0.029, HR 0.36 (0.14–0.93)) [10]. Another study by Ali et al. followed 23 patients with 26 recurrent brain metastases following treatment with SRS for a median of 141 days [14]. While no specific OS or time to progression was reported, they noted that within their cohort, when ≥80% ablation was achieved, no patients progressed. Interestingly, in five patients in which <80% ablation was achieved, post-LITT SRS resulted in zero cases of progression [14]. While this subset analysis is small, it suggests possible synergy between LITT and SRS, as well as a means to rescue a patient in which suboptimal ablation has been achieved. These positive findings should be weighed against the small sample size and short median follow-up time.

The Laser Ablation after Stereotactic Radiosurgery (LAASR) trial was the first multicenter, prospective study of brain metastases and emphasized the importance of achieving complete tumor ablation [15]. The 42-patient cohort consisted of 20 recurrent metastases, 19 patients with radiation necrosis and 3 unknown diagnoses. Of these patients, only 25% of completely ablated tumors progressed, compared with a 62.5% rate of progression when ablation was subtotal, although the analysis was limited to a 6-month follow-up period [15]. In a different study, in a small heterogenous cohort of twenty patients with radiation necrosis deemed inaccessible to open surgery, patients treated with radical ablation volumes (RVAs) presented an increase of >200% (*n* = 4) in pre-LITT lesion volume or an increase of >2 mm in pre-LITT lesion diameter, resulting in improved PFS (*p* = 0.046 and *p* = 0.038, respectively) and OS (*p* = 0.04) compared with a cohort receiving either treatment with subtotal volumetric ablation (STVA), <100% ablation volume (*n* = 5) or total volumetric ablation (TVA; *n* = 11). [16]. Furthermore, STVA was shown to have a significant higher risk of progressive disease (HR 12.4; *p* = 0.004) [16]. A follow-up study by the same group suggested that supra-lesional ablation volumes resulted in the improvement of postoperative Karnofsky performance scores (KPSs) and a greater reduction in perilesional edema, which, although not reported in this study, may make possible a reduction in, or potentially the cessation of, steroid usage [17]. Of note, in their initial study, STVA, TVA and RVA were not shown to influence steroid regimens; however, this is difficult to effectively evaluate in a retrospective analysis [16].

A 2018 multicenter retrospective study of 30 patients with previously irradiated brain metastases was retrospectively conducted. In this cohort, LITT allowed almost 75% of patients to ultimately stop their steroid regimen within 5 weeks postoperatively, with 63% demonstrating a reduction in perilesional edema volume [18]. Further, nearly half of patients showed an improvement of their neurologic symptoms, with 32% demonstrating complete resolution and another 16% showing partial resolution [18].

Hong et al. were the first group to publish a direct comparison of LITT versus craniotomy for the treatment of both recurrent disease and radiation necrosis [19]. Their total cohort consisted of 75 patients, of which 42 had recurrent disease treated with craniotomy (*n* = 26) or LITT (*n* = 16), who had failed radiotherapy. Local control and overall survival were similar between the craniotomy and LITT treatment groups, with both PFS (1-year PFS, 72.2% vs. 61.1%; 2-year PFS, 60.0% vs. 61.1%, respectively; *p* = 0.72) and OS (1-year OS,69.0% vs. 69.3%; 2-year OS, 56.6% vs. 49.5%, respectively; *p* = 0.90) demonstrating no significant differences. In addition to achieving similar PFS and OS, LITT also had a similar perioperative complication rate (35.3% versus 24.4%; *p* = 0.32) and similar rates of steroid cessation (34.8% versus 47.4%; *p* = 0.53) compared to open surgical resection. Traditionally, a tumor diameter of ≥3 cm serves as a maximum cutoff for treatment with stereotactic radiotherapy. Therefore, Hong et al. performed an additional subset analysis stratifying their cohort by maximal diameter (≥3 cm or <3 cm). This stratification did not lead to significantly different PFS outcomes, thus supporting LITT as a viable, non-inferior option also for large recurrent tumors. Among the limitations to this report were the overwhelming majority of supratentorial tumors, with only nine deep-seated tumors, and a lack of distinction between eloquent- and non-eloquent-area tumors.

A large retrospective review of 70 patients aimed to describe performance status outcomes and effects on cause of death in previously irradiated brain metastases with in-field recurrence treated with LITT. Patients were followed for a median of 12 months to determine effect on KPS and distinguish between neurologic versus systemic causes of death [20]. Of the 70 patients, the cause of death was determined in 44 cases, with 20 dying from a neurologic cause and 24 from a non-neurologic cause [20]. Those who died from a neurologic cause had a significantly lower KPS prior to LITT treatment compared with those who died of a non-neurologic cause (70 ± 14.1 versus 78.8 ± 12.6; *p* = 0.04) [20]. It is important to note that three patients that died of a neurologic cause had tumors treated within the thalamus or brain stem, whereas none of the patients with non-neurologic causes of death had similar deep-seated lesions (*p* = 0.049) [20]. Additionally, patients previously treated with whole-brain radiotherapy had a higher rate of neurologic cause of death than non-neurologic cause (50% versus 16.7%, respectively; *p* = 0.02) [20]. Again, a direct comparison to craniotomy or SRS for in-field recurrent disease was not made. However, historically, although inconsistent in the literature, some reports of SRS for recurrent disease had a predominance of neurologic causes of death [21,22]. LITT also offers the ability for biopsy to confirm disease recurrence versus treatment effect and, in the setting of disease recurrence, the ability to perform molecular analyses to aid the multidisciplinary oncology team in the treatment of progressive disease. Tissue analyses are obviously not an option when treating recurrence with radiation therapy.

There are very few studies with the primary aim to evaluate the use of LITT specifically in the treatment of recurrent disease in deep or eloquent areas. Recently there have been several reports and a meta-analysis on the treatment of recurrent posterior fossa tumors with LITT. Several reports regarding the feasibility of LITT for posterior fossa lesions have previously been published in the glioma literature. Traylor et al. were the first to specifically describe and evaluate the use of LITT in recurrent posterior fossa brain metastases (*n* = 8 recurrent, *n* = 5 RN) [23]. Overall, patients tolerated the procedure well without significant complications. The median cavity volume after ablation initially enlarged from 4.66 cc to 6.29 cc; however, at 9 months, the cavity was noted to have regressed to 2.9 cc [23]. Additionally, the peritumoral edema volume was noted to decrease following LITT (12.25 cc to 5.77 cc at 1 month) [23]. Among the eight patients with recurrence, a median PFS of 7 months and a median OS of 40 months were achieved [23]. A larger, subsequent study was published by Ashraf et al. evaluating 44 previously irradiated posterior fossa brain metastases demonstrating in-field recurrence following SRS. While they did not compare the outcomes following treatment with LITT to those in patients treated with craniotomy, the group demonstrated nearly 90% local control following LITT at 12 months [24]. The median PFS (11.5 months) for recurrent lesions was slightly improved compared with the previous report by Traylor et al. [23,24]. While not the focus of this review, data from studies regarding the management of glioma in eloquent areas can be extrapolated for utilization in the management of recurrent brain metastases as they pertain to feasibility and complications. Shah et al. reported an evaluation of 100 consecutive LITT cases. In the report, they identified 11 newly diagnoses glioma and 14 recurrent glioma tumors that were considered deep seated. In newly diagnosed but traditionally inoperable gliomas, a mean extent of ablation of 98% was achieved, compared with recurrent gliomas, in which a 87.5% mean extent of ablation was achieved. All 25 patients were treated with only one reported complication (wound infection) over a mean 5.6-month (new glioma) and a mean 7.3-month (recurrent glioma) follow-up period [25]. Additional case reports reported the use of both staged LITT procedures for large-volume tumors and awake LITT to ensure the avoidance of eloquent areas [26,27].

While the data on the management of recurrent posterior fossa brain metastases with LITT are limited, the available data consistently report its safety and feasibility in the appropriate patient. However, due to the paucity of research, its efficacy compared to open craniotomy is unclear. As the management of recurrent disease is the focus of this manuscript, it is important to consider the well-described risk of leptomeningeal dissemination of disease following the surgical management of posterior fossa brain metastases. Siomin et al. observed the development of leptomeningeal disease in nearly 50% of patients undergoing the surgical resection of posterior fossa metastases and in 6.5% of posterior fossa lesions treated with stereotactic radiosurgery [28]. Comparatively, in a multi-institutional analysis of LITT for posterior fossa lesions, including a review of 16 studies with 150 patients with varying malignancies, there were zero reported cases of LMD after LITT [29].

### 4.3. Limitations of LITT

Laser interstitial thermal therapy is a powerful addition to the neurosurgeon’s armamentarium. Its role in the management of neuro-oncologic pathologies is not well understood, but evidence suggesting the safe and effective utilization of this technology is mounting. However, still in its infancy, the data are sparse. The clinical evaluation of LITT for recurrent brain metastases is limited to a handful of small retrospective, single-center analyses. Further, the studies that are available are typically technical descriptions of the authors’ experience, describing the extent of safe ablation, tumor response to ablation and, in some reports, the ability to successfully and efficiently wean off or reduce steroid utilization. Unfortunately, the question of craniotomy versus LITT still remains unanswered. Hong et al.’s remains the first and only study with a reasonable sample size directly comparing craniotomy versus LITT, albeit using a retrospective method in a heterogenous cohort with unavoidable patient selection biases. Nonetheless, their observations certainly suggest the importance of further evaluation. It appears that LITT may potentially be equally efficacious as craniotomy for recurrent brain metastases with similar and durable rates of PFS in the appropriately selected patient.

### 4.4. Current Research and Future Directions

As displayed by this manuscript, data regarding LITT for recurrent metastatic disease are sparse. Currently, there are two active clinical trials studying LITT for brain metastases, of which only one (Duke University and Wake Forest University) compares the outcomes for recurrent brain metastases treated with LITT versus LITT plus hypofractionated SRS. The other trial evaluates the response to LITT treatment with F18 fluciclovine PET scan (MD Anderson). Further studies of LITT in the treatment of recurrent brain metastases must prospectively compare craniotomy versus LITT and SRS versus LITT to better understand optimum treatment modalities for recurrence. A propensity-based score would be ideal in determining the appropriate patient to treat with LITT over craniotomy or SRS. Ultimately, the best clinical practice may require the development of an algorithm that helps to select the ideal patient for open resection versus LITT versus radiation, but at this time and with the available data, such a determination is not possible.

## 5. Conclusions

The use of laser interstitial thermal therapy in the treatment of recurrent brain metastases is in its infancy. Neurosurgeons and neuro-oncologists currently utilize it as a means for a minimally invasive approach to recurrent disease, particularly in patients that are unlikely to tolerate craniotomy for resection or those with deep-seated or eloquent areas that are less amenable to open resection. The data presented in this review suggest that the use of LITT among this patient population is both feasible and safe. It appears to be effective in most reports; however, there is a severe lack of class 1 and 2 evidence to support the use of LITT instead of craniotomy for resection. Thus, it is important to pursue further prospective and randomized evaluations of LITT compared to craniotomy in matched cohorts to best determine the role of LITT in the management of recurrent brain metastases.

## Figures and Tables

**Table 1:** Summary of selected studies.

Author	Year	#Total Lesions	#Total Recurrent Lesions	Location(s) Supratentorial Infratentorial Deep Seated (Respectively)	PFS	OS	Follow Up (Median, Months)
Ali et al.	2016	23	26	17 1 8	100% when ≥80% ablation	NA	4.7
Ahluwalia et al.	2018	42	20	Includes all, but unable to distinguish	74%	72%	6.5
Traylor et al.	2019	13	8	8 infratentorial only	7 m	40 m	NA
Kamath et al.	2017	25	25	Includes all, but unable to distinguish	NR	17.2 m	9.8 m
Ashraf et al.	2020	60	44	44 infratentorial only	11.5 m (89.7% at 12 m)	NA	NA
Salehi et al.	2018	25	25	22 1 2	6.3 m	13.3 m	16.1 m
Hong et al.	2019	75	16	Includes all, but unable to distinguish	54.7% at 12 m	NA	NA
Kaye et al.	2020	97	97	73 8 4	5.6 m	11.9 m	12.0 m
Dabeco et al.	2021	19	12	Includes all, but unable to distinguish	7 m	25 m	10 m
Chaunzwa et al.	2018	30	30	Includes all, but unable to distinguish	83% overall	26.1% at 12 m	5.6 m
Overall		412	303	112 62 14	7.5 m	21.5 m	6.5 m

NA: not applicable; NR: not reached; m: months.
